# Investigations of a Rabbit (*Oryctolagus cuniculus*) Model of Systemic Lupus Erythematosus (SLE), BAFF and Its Receptors

**DOI:** 10.1371/journal.pone.0008494

**Published:** 2009-12-30

**Authors:** Jiahui Yang, Richard Pospisil, Satyajit Ray, Jacqueline Milton, Rose G. Mage

**Affiliations:** Laboratory of Immunology, National Institute of Allergy and Infectious Diseases, National Institutes of Health, Bethesda, Maryland, United States of America; University of Miami, United States of America

## Abstract

B-cell activation factor belonging to the tumor necrosis factor family (BAFF) is a major contributor to survival of B lymphocytes during development and maturation. A relationship between circulating BAFF levels and disease activity has been reported in patients with the autoimmune disease Systemic Lupus Erythematosus (SLE). Clinical trials targeting BAFF or its receptors are currently in progress. In order to further characterize a rabbit (*Oryctolagus cuniculus*) model of SLE, we investigated the expression of BAFF and its receptors in non-inbred, pedigreed rabbits derived from breeding and selection based on autoantibody responses. We immunized rabbits related to previous groups that developed autoantibodies and inflammatory responses after immunizations with peptides synthesized on multiple antigen-branched polylysine backbones. Blood and sera collected before immunization and after boosts were used for health monitoring, analyses of serum autoantibody responses by ELISA and immunofluorescence. Peripheral blood mononuclear cells (PBMC) were studied by flow cytometry and were the source of mRNA for quantitative PCR analyses. We hypothesized that BAFF mRNA expression and serum BAFF levels measured indirectly through BAFF receptor binding might increase in autoantibody-producing rabbits. Immunized rabbits developed elevated levels of leucocyte populations, anti-nuclear, anti-dsDNA and other autoantibodies. BR3 mRNA levels in total PBMC decreased and BAFF levels remained low and unchanged in most immunized rabbits. By flow cytometry, percentages of BAFF positive cells decreased. Percentages of transmembrane activator and CAML interactor (TACI) decreased in most rabbits from all the immunized groups. The rabbit is an important model for human autoimmune and infectious diseases, and a high quality draft rabbit genome assembly was recently completed. Human disease models developed in non-inbred pedigreed animals are better able to reflect the complexities of diseases such as SLE with familial patterns of inheritance. Although no consistent pattern of elevated expression of BAFF mRNA or protein was found in the rabbits studied, the data collected and reported here build upon previous data to refine understanding of a rabbit model of SLE.

## Introduction

Systemic Lupus Erythematosus (SLE) is a complex autoimmune disease with considerable heterogeneity in clinical manifestations and disease course. Although multiple immunologic abnormalities contribute to the development of SLE, it is generally accepted that B cells play key roles in disease pathogenesis. B cells are responsible for tissue damage both by generating autoantibody-producing cells and through other indirect mechanisms that may break T cell tolerance. Understanding the factors that control the survival of these pathological B cells is essential for elucidating the pathogenesis of SLE (reviewed in [1], [2]) and providing new targets for SLE therapy (reviewed in [3], [4]).

Our laboratory described a model of SLE in NIAID-pedigreed non-inbred rabbits [5], based on earlier reports of James et al. [6] that Multiple Antigen Peptide (MAP-8) immunogens elicited lupus-like autoantibody responses. Their immunogen carried eight copies of a peptide sequence derived from EBV EBNA1 protein with sequence similarity to spliceosomal SmB/B′ protein Smith (Sm) autoantigen. We confirmed their observation of autoantibody responses following immunization and boosting with the same peptide antigen and extended the work by immunizing with a peptide from rabbit N-methyl-D-aspartate receptor NR2b (GR) [5]. Cross reactivity of some anti-dsDNA autoantibodies with this important receptor led to the suggestion that such cross reactive anti-dsDNA antibodies play a role in neuropsychiatric manifestations of SLE [7], [8]. All of the rabbits produced peptide-specific antibodies and some also produced autoantibodies including anti-dsDNA and anti-nuclear antibodies resembling those found in human lupus patients. Two rabbits experienced seizures and one developed nystagmus [5]. Heterogeneity of responses was confirmed in further investigations by Puliyath et al [9] who used the GR-MAP-8 peptide to immunize animals selectively bred from earlier responder rabbits and provided evidence for genetic contributions to autoantibody profiles in the rabbit model of SLE.

B-cell activating factor (BAFF; also termed BLyS, CD257, TNFSF13B, TALL-1, and zTNF4) is a potent B cell survival factor mainly expressed by myeloid cells and monocyte-derived dendritic cells. Receptors with distinct developmental and functional roles are the BAFF receptor (BR3 also termed BAFF-R), the transmembrane activator and calcium-modulator and cyclophilin ligand interactor (TACI) and the B cell maturation antigen (BCMA) (reviewed in [10]). TACI and BCMA bind both BAFF and APRIL, another TNF ligand superfamily member. TACI plays a role in negatively regulating mature B-cell homeostasis, but TACI also plays a role in T-independent B-cell responses and class switch recombination [11]. Recent reports suggest a role of BCMA in controlling the lifespan of long-lived plasma cells [12]. The numbers of mature B cells in secondary lymphoid organs, baseline serum Ig levels and Ig responses to T-cell-dependent and -independent antigens, are reduced in mice that cannot express BAFF. Transgenic mice that over express BAFF develop autoimmune disease resembling human lupus. Although T cells are not required for the disease development in this mouse model, innate immune signals through MyD88 are required for B cell activation and production of autoantibodies [13].

Disease activity in human lupus patients has been reported to correlate with serum BAFF levels (reviewed in [14]) and levels of expression of BAFF, TACI and BR3 mRNA in PBMC of patients with SLE were reported to be significantly elevated [15]. Clinical trials targeting BAFF or its receptors are currently in progress [16]; (also reviewed in [17], [18]). We chose to investigate BAFF and its receptors in the rabbit SLE model because of their potential as therapeutic targets for human SLE and the reported correlations of disease activity with elevated serum BAFF protein [14] and BAFF mRNA in PBMC [15]. On July 20, 2009, Human Genome Sciences and GlaxoSmithKline announced that a placebo-controlled BLISS-52 study of BENLYSTA™ (belimumab, formerly LymphoStat-B®) met the primary endpoint in the first of two Phase 3 trials in patients with serologically active SLE. See: http://www.hgsi.com/latest/human-genome-sciences-and-glaxosmithkline-announce-positive-phase-3-study-results-for-benl-2.html.

Before initiating this investigation of the role of BAFF and its receptors in the rabbit model of SLE, we first studied expression and localization rabbit BAFF and its specific high affinity receptor BR3 in cells and tissues of the normal rabbit immune system [19]. We found that mRNA for both BAFF and BR3 was detectable in normal rabbit peripheral blood mononuclear cells (PBMC). BAFF message was detectable in CD14^+^ macrophage/monocyte populations as well as some CD14^−^ cell populations from peripheral blood and spleen. We found high levels of mRNA for BR3 in normal unstimulated IgM^+^ B cells but they did not have detectable BAFF message. Since surface BAFF was detected by flow cytometry and immunohistochemical staining even though no mRNA was detected, we suggested that soluble BAFF was probably bound to BAFF receptors on IgM^+^ B cells. We also detected BAFF by immunohistochemical studies of developing neonatal rabbit appendix and suggested that BAFF could play a role in early stages of normal rabbit B-cell development [19].

Based on this initial groundwork, we set out to investigate BAFF and its receptors during B-cell activation and autoantibody production in rabbits derived from selective breeding to develop a reproducible rabbit model of SLE. Flow cytometry was used to detect BAFF, BR3 and TACI on PBMC prior to and after immunization and boosting in order to seek associations of levels of BAFF, BR3 and TACI with autoantibodies detected in serum and hematological changes in blood. Quantitative PCR was used to detect expression of BAFF and BR3 in PBMC prior to and after immunization and boosting. In addition, β2-microglobulin (B2M) mRNA expression was measured because it was found in earlier studies to be elevated in expression in some lupus rabbits (Rai et al. ms in preparation). Increased levels of B2M reflect elevated levels of class I MHC molecules in activated PBMC. This study broadens our knowledge of the expression of this important member of the TNF superfamily in a non-inbred pedigreed rabbit model of SLE where BAFF may specifically regulate B- lymphocyte proliferation and survival.

## Materials and Methods

### Animals

The animal studies described here were reviewed and approved by the animal care and use committees of NIAID/National Institutes of Health, Bethesda, MD (animal study protocol LI-6) and of the Spring Valley Laboratories (Woodbine, MD), where the NIAID allotype-defined rabbit colony was housed. Rabbits of the experimental group were relatives and descendants bred and developed from the previous lupus experimental groups, based on autoantibody responses. The animals' designations, sexes, ages at the start of the experiment, immunoglobulin allotypes including VHa encoded at the *Igh* locus and C*κ*b encoded at the light chain (Cκ1) locus [20] are summarized in Table 1. The rabbits were divided into four groups (A, B, C, and D) to allow handling of blood for preparation of PBMC for FACS and mRNA isolation. One control rabbit (CF) in group C died and was not replaced.

**Table 1:** Immunized rabbits, sexes, Ig allotypes and ages.

Group	Rabbit ID	Rabbit no.	Sex	Allotype	Age (In months)
A	GR72	UA345-5	F	a2R3/2R3, b4/9k	13.5
	GR73	UA269-3	F	a1/1, b4/5	8.8
	BB74	6YY328-4	M	a1/1, b5/9	24.9
	BB75	2YY125-6	M	a1/2, b9k/9k	18.2
	CF1	6YY328-3	M	a1/1, b5/9	24.9
	CF2	1UA161-1	M	a1/1, b9/9	7.4
B	GR76	2YY119-9	F	a1/1, b9/9	18.1
	GR77	UA269-1	M	a1/1, b4/5	8.8
	BB78	YY118-6	M	a1/1, b9/9	18.0
	BB79	1UA161-2	M	a1/1, b9/9	7.4
	CF3	1YY125-4	M	a2/2, b4/9k	18.2
	CF4	2YY125-4	M	a2/2, b5/9k	18.2
C	GR80	XA345-1	F	a1/2, b9/9k	6.3
	GR81	2UA14-2	F	a1/1, b5/9	7.4
	BB82	XA346-2	M	a1/1, b9/9	6.3
	BB83	2UA14-3	F	a1/1, b5/9k	7.4
	CF5	XA345-2	F	a1/2, b9/9k	6.3
D	GR84	XA234-6	F	a1/2, b5/9	6.3
	GR85	XA346-1	M	a1/1, b9/9	8.9
	BB86	XA234-2	M	a1/2, b5/9	5.3
	BB87	3XA203-2	M	a1/ali, b5/9	4.2
	CF6	2XA344-2	F	a1/1, b9/9	8.8
	CF7	1XA344-1	M	a1/1, b9/9	8.8

### Pedigree analysis

Breeding information and experimental records on the breeding colony were entered into a custom designed 4^th^ Dimension relational database (Tiller systems, Brunswick, MD). Information from the database was exported into Pedigree-Draw for generation of pedigrees (Jurek Software, Cottage Grove WI).

### Antigens

The peptide sequence (DEWDYGLP) from the extracellular domain of rabbit neuronal postsynaptic glutamate receptor N-methyl-D-aspartate (NMDA) NR2b chain (peptide GR) was synthesized on a multiple antigen-branched polylysine (multiple antigen peptide) (MAP) backbone (BB). BB-MAP-8 without peptide was synthesized as control immunogen. In addition, adjuvant control rabbits (CF) received initial Complete Freund's Adjuvant (CFA) followed by boosts with Incomplete Freund's Adjuvant (IFA) emulsified with the same diluent used for the antigens. All synthesized structures were analyzed by HPLC (National Institutes of Health Peptide Synthesis and Analysis Unit, Research Technology Branch, Rockville, MD).

### Immunization

Each rabbit received initial subcutaneous (s.c.) injection of either GR-MAP-8 (GR rabbits), control BB (BB rabbits) (0.5 mg/0.5 ml in borate-buffered saline pH 8.0 (BBS), emulsified with 0.5 ml of CFA), or control borate buffered saline, pH 8.0 (BBS) (0.5 ml of BBS, with 0.5 ml of complete Freund's adjuvant (CFA) (CF rabbits). Rabbits received either 3 or 5 boosts given s.c. at 3-wk intervals with the same antigen concentration or BBS) emulsified with incomplete Freund's adjuvant (IFA). After the final boost in IFA, rabbits were injected intravenously (i.v.) 3 weeks later with the same antigen concentration or BBS. Sera and heparinized whole blood cells were collected immediately before immunization (pre-immune) and 1 wk after each boost (post-boost). Sera were aliquoted and stored at −20°C for assay. A sample of whole blood was collected for complete blood cell and chemistry analysis, and the remaining blood used for PBMC isolation for flow cytometry analysis and mRNA. Blood for chemistry, hematology and PBMC for mRNA analyses was collected a week later, and rabbits euthanized 7 or 8 days after the final i.v. boost.

### Clinical assessment

The rabbits were regularly monitored for clinical signs of disease and subjected to periodic health examinations. Whole blood and sera collected after boosts were sent to a veterinary diagnostic laboratory (Antech Diagnostics) for complete blood counts and chemistry panels as previously described [5], [9].

### ELISA for anti-peptide antibodies, anti-dsDNA and autoantibodies to nuclear antigens

Serum antibody responses to the GR-MAP-8 and control immunogens were measured by standard solid-phase ELISA. Polystyrene 96-well plates (Corning; catalog no. 3590) were coated with 50 µl/well of GR-MAP-8 at 10 µg/ml in bicarbonate buffer (pH 9.6) and incubated overnight at 4°C. Plates were washed three times with PBS (pH 7.2) containing 0.1% Tween 20 and blocked with 100 µl of blocking solution for 1 h at 37°C (Quality Biologicals). Wells were then incubated 1 h at 37°C with 50 µl/well of 1/1024-diluted sera, washed five times, incubated for 1 h at 37°C with 50 µl of a 1/2000 dilution (0.4 ng/µl) of affinity-purified HRP-conjugated goat anti-rabbit IgG (H + L) secondary antibody (Jackson ImmunoResearch Laboratories), developed with 3,3′, 5,5′-tetramethylbenzidine (TMB) (INOVA Diagnostics), and the resulting OD read at 450 nm. Commercially available human diagnostic kits (INOVA Diagnostics) were used to assay serum antibodies to calf thymus dsDNA and autoantibodies to nuclear antigens (ANA), and components (Sm and RNP) as previously described [9]. Briefly, 100 µl of rabbit sera diluted 1/41 in the proprietary sample diluents were added to antigen-coated wells and incubated for 30 min at RT. Wells were then washed, incubated for 30 min at RT with 1/8000 diluted HRP-conjugated goat anti-rabbit IgG Fc (Jackson ImmunoResearch Laboratories), and developed with TMB for reading OD at 450 nm.

### Antibodies and reagents for flow cytometry

Surface staining for BAFF was determined using biotin-conjugated goat anti-human BAFF polyclonal antibody that cross-reacts with rabbit BAFF; staining for TACI used biotin-conjugated goat anti-human TACI polyclonal antibody that cross-reacts with rabbit TACI (Antigenix, America, Inc.). Surface staining of BR3 was detected using purified goat anti-human BR3 antibody that is cross-reactive with rabbit BR3 (R&D systems) [19]. Also used were FITC-labeled mouse anti-rabbit CD14 (clone K4) (Antigenix, America, Inc.), FITC-labeled goat anti-rabbit IgM (Southern Biotechnology Associates), biotin-labeled donkey anti-goat IgG, biotin-labeled goat IgG, normal goat IgG (Jackson ImmunoResearch Laboratories, Inc), and FITC-labeled mouse IgG2a (BD Pharmingen). Biotinylated antibodies were visualized by PE-conjugated streptavidin (Jackson ImmunoResearch Laboratories, Inc).

### Detection of anti-nuclear antibodies (ANA) by indirect immunofluorescence

Commercially available slides coated with fixed HEp-2 cells (Antibodies, Inc.) were incubated with rabbit antisera diluted 1/20 in 5% goat serum (Jackson ImmunoResearch Laboratories) for 30 min at room temperature (RT). ANA binding was detected by fluorescence microscopy following 30-min incubation at RT with 12.5 ng/µl FITC-goat anti-rabbit IgG Fc (Southern Biotechnology Associates). Secondary antibody against IgG was selected to avoid unwanted background fluorescence and positive results of doubtful clinical importance. Fluorescent binding patterns were compared with pictures in von Muhlen and Tan [21] and with reference pictures provided with the Antibodies, Inc. kit.

### Cell isolation

Viable PBMC were isolated from heparinized blood by density gradient centrifugation on Lympholyte-Mammal (Cedarlane Laboratories Limited). This permitted recovery of lymphocytes and monocytes but eliminated most red cells and granulocytes. After isolation, cells were immediately placed on ice for flow cytometry assays or placed into TRIzol™ reagent (Invitrogen) for RNA isolation.

### Flow cytometry

Purified PBMCs were stained using standard flow cytometric methods. Briefly, cells were incubated on ice for 40 min with primary antibody before washing twice with cold PBS containing 1% FCS, then subsequent incubation with various secondary reagents or secondary antibodies [19]. For BR3 detection, a biotinylated donkey anti-goat IgG was used as secondary antibody. Biotinylated antibodies were visualized by PE-conjugated streptavidin (Jackson ImmunoResearch Laboratories, Inc). After washing, cells were analyzed using a FACS-Calibur flow cytometer (BD Pharmingen) and FlowJo analytical software (Tree Star). Cells were gated on side scatter×forward scatter (SSC×FSC) profiles to include both small and large lymphocytes, as well as monocytes but exclude red blood cells and granulocytes; dead cells were excluded by propidium iodide staining. Rabbit IgM^+^ B cells were detected by FITC-conjugated goat anti-rabbit IgM (Southern Biotechnology Associates).

### Detection of expression levels of BAFF, BR3 and other mRNA

Viable PBMC isolated from heparinized blood by density gradient centrifugation on Lympholyte-Mammal (Cedarlane Laboratories Limited) were immediately placed in TRIzol reagent after isolation, homogenized and filtered with Qiashredder column (Qiagen). Total RNA was isolated by RNeasy spin column (Qiagen) and precipitated with ethanol. First-stand cDNA was synthesized using the SuperScript.

First-strand synthesis kit (Invitrogen). Quantitative PCR to determine the relative expression levels of BAFF, BR3 and other mRNAs was performed on 9700HT Sequence Detection System (Applied Biosystems). Synthesized cDNA from isolated PBMCs was directly used as template for real-time PCR by using TaqMan 2× PCR Master Mix Reagents Kit (Applied Biosystems). Relative expression levels of an additional mRNA for rabbit β2-microglobulin (B2M) found in earlier studies (Rai et al. ms in preparation) to be elevated in expression in some lupus rabbits was also measured. The primers, probes and PCR conditions used are shown in Table 2. Each sample from three independent experiments was run in duplicate. The unit number showing relative mRNA levels in each sample was determined as a value of mRNA normalized against peptidylprolyl isomerase A (PPIA). Previous studies showed uniform expression of PPIA in total WBCs from both normal and treated rabbits (Rai et al. ms in preparation).

**Table 2:** Primers and probes for quantitation of mRNA by Q-PCR for rabbit BAFF, BR3, PPIA control and β2 microglobulin (B2M).

A. BAFF and BR3^a^	
BAFF forward	5′ TGGTCAAAGAAACTCGGGTACTT 3′
BAFF reverse	5′ TGTTTTGAATGCAACGGAACA 3′
BAFF probe	5′ TCATATACGGTCAGGTCTT 3′
BR3 forward	5′ CGGGACGGAGACCAGGA 3′
BR3 reverse	5′ TGAGTTGGGAGCTGTGGCA 3′
BR3 probe	5′ AGTCCCTGGATGATGTCA 3′
B. PPIA control^a^	
PPIA forward	5′ CAACACAAATGGCTCCCAGTT 3′
PPIA reverse	5′ CATGGCTTCCACAATGCTCAT 3′
PPIA probe	5′ ATCTGCACTGCCAAGAC 3′
C. B2M^a^	
B2M forward	5′ TTGTTCCCCTGCCTGGAGT 3′
B2M reverse	5′TGGATGACGAGAGTACACTTGAACAT 3′
B2M probe	5′ CCAGCGTGCTCCG 3′

a The total volume of PCR reaction was 25 µl and the PCR conditions were as follows: 50°C for 2 min, 95°C for 10 min, followed by 40 cycles of 95°C, 15 s for denaturation and 60°C, 1 min for annealing and extension.

### Statistical analysis

Student's t test, Pearson's correlation analysis and ANOVA procedures were performed using SAS v8.0, JMP-7 and Prism software to examine associations of BAFF, BR3, and TACI measurements with antibody responses and hematological data.

## Results

### Clinical findings and leukocyte responses

As in the previously studied rabbits that developed autoantibodies [5], [9], no evidence of kidney damage or pathology was seen in the present group. However, as observed in the 5^th^ group [9], hematological profiles suggested development of chronic inflammatory responses. Supporting online Table S1 summarizes observed changes in total White Blood Cell (WBC), Monocyte, Neutrophil, Eosinophil and Basophil counts after immunization. Values in bold face are above the normal reference range shown in the footnote to the table. With the exception of two Freund's immunized control rabbits (CF3 and CF4), rabbits developed higher WBC counts by the third or fifth boost with many above the normal reference range. Total WBC counts were elevated above the normal reference range (4–10×10^3^/µl) in two littermates (BB 74 and CF1) prior to immunization (pre) and became further elevated after the 3^rd^ boost. The mean WBC counts (×10^3^/µl) in all rabbits prior to immunization were 7.30+/−1.71 (SD) and rose to 11.40+/−3.17 and 11.60+/−3.12 after the third and fifth boosts. Lymphocyte numbers generally remained within the normal reference range (1.2–7×10^3^/µl) but there were elevations in neutrophils (2.0+/−0.79 in pre and 5.9+/−2.23, 5.1, +/−2.02 after 3^rd^ and 5^th^ boosts) and monocytes (0.26+/−0.13 in pre and 0.65+/−0.29 and 0.57+/−0.25 after 3^rd^ and 5^th^ boosts). Some eosinophil and basophil counts also increased with a few shown in bold face in Table S1 exceeding the normal reference ranges (0–0.10;0–0.50). These results confirm and extend those previously reported [9] that raised the possibility that among these selectively bred pedigreed animals, CFA followed by IFA could generate the elevated leukocyte response we found after immunization with GR-MAP-8 or the branched BB-MAP-8 without additional peptide. The CF group was added to the present study because we had not previously investigated the role of CFA followed by IFA. We conclude that in some of these selectively bred rabbits, elevated leukocyte response typical of chronic inflammatory responses but not of human SLE can occur due to the immunization protocol using CFA and IFA.

### Anti-peptide and autoantibody responses

**Figure 1:**
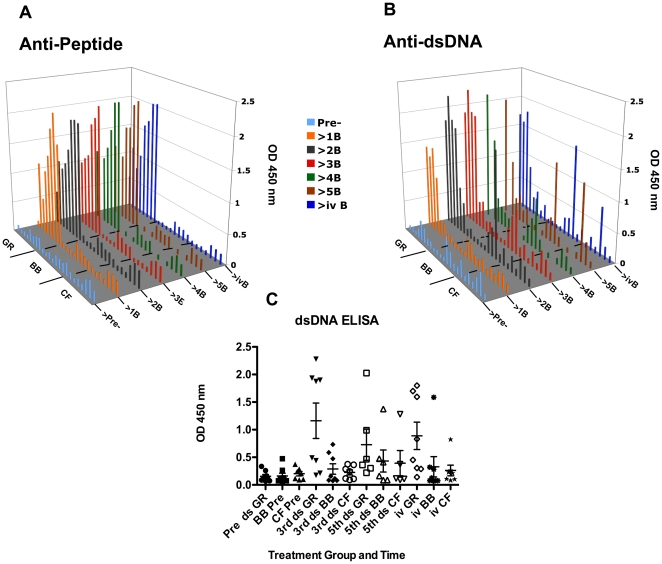
ELISA measurements of anti-peptide and anti-dsDNA responses. In panels A (anti-peptide B) and B (anti-dsDNA), the pre-immune OD 450 nm values are shown in light blue and the responses after each boost are shown in distinct colors. The low pre-immune values were not subtracted from the post-immune values shown. C. Mean responses of GR-, BB- and CF- immunized rabbits showing high and low anti-dsDNA responders (ds) among GR-MAP-8 immunized rabbits. Each symbol represents an individual rabbit serum tested prior to immunization (Pre ds), after the 3^rd^, 5^th^ and intravenous (iv) boosts.

Table 3 contains primary data obtained by Q-PCR, ELISA and ANA indirect immunofluorescence. Figure 1 summarizes pre-immune and post-immune measurements of anti-peptide and anti-dsDNA responses. In panels A and B, the pre-immune OD 450 nm values are shown in light blue and the responses after each boost are shown in distinct colors. The low pre-immune values were not subtracted from the post-immune values shown. Four of the eight rabbits that were immunized with GR-MAP-8 developed high anti-dsDNA by the third boost and four developed lower responses (Figure 1C). A marked increase in anti-dsDNA was measured in one animal that received MAP-8 backbone (BB86) starting after the second boost, and one animal that received only CFA followed by IFA (CF5) developed anti-dsDNA after the 5^th^ boost.

**Table 3:** Summary of mRNA expression data and autoantibody responses.*

	Q-PCR									ELISA															IFA		
	BR3			BAFF			B2M			dsDNA			Peptide			Sm			RNP			ANA			ANA		
Rabbit	Pre	3rd	5th	Pre	3rd	5th	Pre	3rd	5th	Pre	3rd	5th	Pre	3rd	5th	Pre	3rd	5th	Pre	3rd	5th	Pre	3rd	5th	Pre	3rd	5th
UA345-5(GR72)	81.01	*18.51*		6.06	**2.16**		44	**177.33**		0.067	**1.87**		0.10	**0.75**		0.15	**0.43**		0.22	**0.47**		1.13	**1.68**		1	1	1
UA269-3(GR73)	35.02	*16.00*	*24.25*	1.69	1.68	2.11	29.42		*1*	0.086	**2.28**	**2.03**	0.07	**1.09**	**1.22**	0.57	0.60	0.57	0.56	0.62	0.56	0.75	**1.15**	**1.22**	2	**3**	**3**
2YY119-9(GR76)	27.47	*15.14*		2.08	2.64		78.86	**188.97**		0.074	**1.94**		0.09	**1.07**		0.20	**0.63**		0.22	**0.49**		0.47	**0.90**		1	**2**	
UA269-1(GR77)	29.86	**32.90**	*26.35*	1.42	2.22	2.16	55.39		*55.92*	0.161	**1.90**	**0.99**	0.07	**1.19**	**1.10**	0.15	**0.34**	**0.25**	0.27	**1.05**	**0.57**	0.47	**0.77**	**0.98**	1	**3**	**4**
XA345-1 (GR80)	28.05	*25.81*	**31.56**	1.87	2.25	2.19	39.68		*32.45*	0.334	**0.44**	0.36	0.22	**1.27**	**1.63**	0.12	0.22	0.17	0.40	**1.04**	**0.51**	0.37	0.37	0.38	1	1	1
2UA14-2(GR81)	33.13	*18.77*	*24.76*	1.37	1.47	2.08	30.1		**74.91**	0.26	**0.49**	**0.47**	0.15	**1.82**	**1.66**	0.12	0.20	0.17	0.13	0.18	0.20	0.30	**0.88**	**1.28**	1	1	1
XA234-6 (GR84)	17.63	**30.27**	**21.41**	0.71	**2.71**	**2.28**	28.43		28.42	0.126	0.17	0.22	0.20	**1.95**	**2.07**	0.29	0.31	0.29	0.22	0.23	0.22	0.38	**0.76**	**0.87**	−1	**1**	**1**
XA346-1 (GR85)	43.11	*39.40*	43.11	1.19	**2.83**	2.03	10.6		**27.6**	0.128	0.21	**0.30**	0.22	**2.17**	**2.16**	0.25	0.35	0.25	0.91	**2.48**	**1.98**	0.47	**1.26**	**1.37**	−1	**4**	**4**
6YY328-4(BB74)	30.48	*25.28*		2.99	**4.08**		90.56		*65.99*	0.07	0.08		0.08	0.08		0.18	0.19		0.19	0.24		2.02	1.89		1	1	
2YY125-6(BB75)	41.07	*26.54*	*9.65*	2.22	2.00	1.68	6.89	**42.37**		0.09	0.11	0.09	0.07	0.07	0.08	0.22	0.31	0.23	0.19	0.23	0.21	0.52	**0.82**	**0.63**	−1	**3**	1
YY118-6(BB78)	50.56	*34.54*		1.79	2.38		43.86	*35.75*		0.07	0.07		0.12	0.12		0.14	0.17		0.65	0.65		1.61	**1.74**		1	**2**	
1UA161-2(BB79)	63.12	*38.59*	*23.59*	1.39	1.91	1.45	36.62		*20.81*	0.07	0.08	0.09	0.07	0.07	0.07	0.14	0.16	0.24	0.17	**0.33**	**0.36**	0.29	**0.52**	**0.41**	1	1	1
XA346-2 (BB82)	37.79	*30.27*	*21.41*	1.40	2.07	**3.34**	63.41		*32.26*	0.48	0.48	0.48	0.18	**0.31**	0.24	0.16	**0.28**	**0.32**	0.16	0.21	0.20	0.51	**1.61**	**1.42**	1	**3**	**3**
2UA14-3(BB83)	29.86	29.65	*23.10*	0.91	**2.48**	**2.57**	38.11		**47.46**	0.26	**0.59**	**0.41**	0.17	0.25	0.17	0.11	**0.23**	**0.29**	0.17	**0.29**	0.22	0.44	**0.66**	**0.64**	1	**3**	**3**
XA234-2(BB86)	34.78	**56.49**	**50.91**	1.35	2.25	**3.84**	16.38		**20.21**	0.12	**0.73**	**1.37**	0.16	0.19	0.17	0.18	0.28	0.18	0.19	0.28	0.20	0.26	0.29	**0.36**	−1	**1**	**1**
3XA203-2(BB87)	52.71	*45.57*	**60.13**	0.71	1.29	1.48	13.38		**20.34**	0.13	0.16	0.16	0.22	0.22	0.22	0.16	0.21	0.20	0.16	0.22	0.18	0.36	**1.61**	**1.06**	−1	−1	**1**
6YY328-3(CF1)	66.72	*31.34*		2.00	**3.14**		58.33	*17.59*		0.29	0.29		0.13	0.13		0.19	0.19		0.24	0.24		1.11	**1.48**		1	**2**	
1UA161-1(CF2)	11.96	**28.05**	**20.39**	1.07	1.39	**2.31**	59.9		*8.75*	0.09	0.09	0.09	0.08	0.08	0.08	0.20	0.28	0.26	0.18	0.22	0.20	0.76	**0.99**	**0.92**	1	**2**	1
1YY125-4(CF3)	63.56	*51.63*		1.47	2.31		29.98	**38.42**		0.21	0.24		0.13	0.13		0.13	0.19		0.14	0.15		1.12	1.12		1	**3**	
2YY125-4(CF4)	36.50	*20.53*	*18.25*	1.58	1.77	1.75	16.88		*5.1*	0.12	0.12	0.12	0.11	0.11	0.11	0.26	0.26	0.26	0.23	0.26	0.23	0.76	0.87	0.76	1	1	1
XA345-2 (CF5)	16.91	*14.93*	17.63	1.55	1.83	**3.78**	36.87		**50.38**	0.24	**0.36**	**1.28**	0.24	0.24	0.26	0.12	0.14	0.13	0.16	0.17	0.16	0.54	0.58	0.59	−1	−1	**1**
2XA344-2 (CF6)	20.39	*15.24*	**21.56**	1.72	**3.01**	**3.23**	27.06		**38.58**	0.37	0.37	0.37	0.28	0.28	0.28	0.40	**0.58**	0.49	0.31	**0.44**	0.33	1.96	1.96	1.96	−1	**1**	**1**
1XA344-1 (CF7)	30.48	**34.54**	**31.56**	1.85	2.58	2.62	18.2		**48.57**	0.10	0.11	0.11	0.18	0.25	0.18	0.20	0.24	0.23	0.17	0.25	0.20	0.21	**0.31**	**0.30**	−1	**1**	−1

* Bold shows values of at least 0.1 (for ELISA) or 1 [for Q-PCR and ANA (IFA)] above the pre-immune values. Italics show values of at least 1 (for Q-PCR) below pre-immune values.

**Figure 2:**
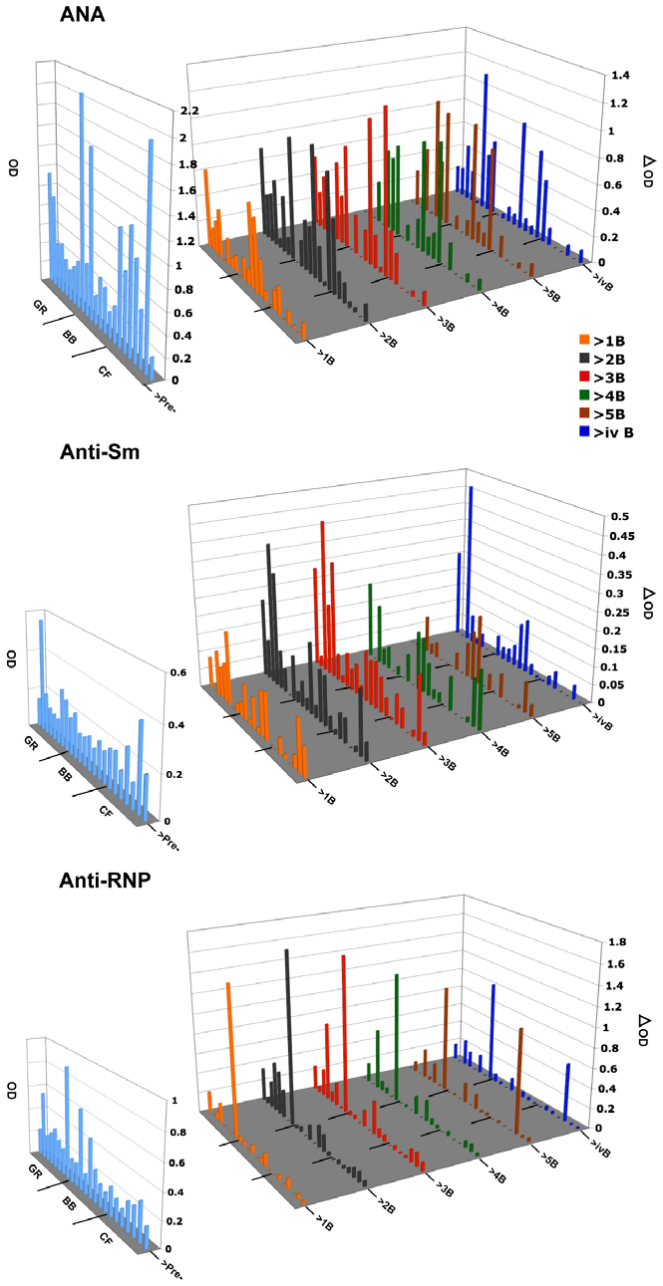
ELISA measurements of other autoantibody responses. The pre-immune OD 450 nm values found by ELISA for anti-nuclear antibodies (ANA), anti-Sm and anti-RNP are shown separately in light blue because of the high levels of antibodies in some rabbits prior to immunization. All post-immunization responses are given as ΔOD (the OD 450 in post-boost sera minus the OD 450 in pre-immune sera).

**Figure 3:**
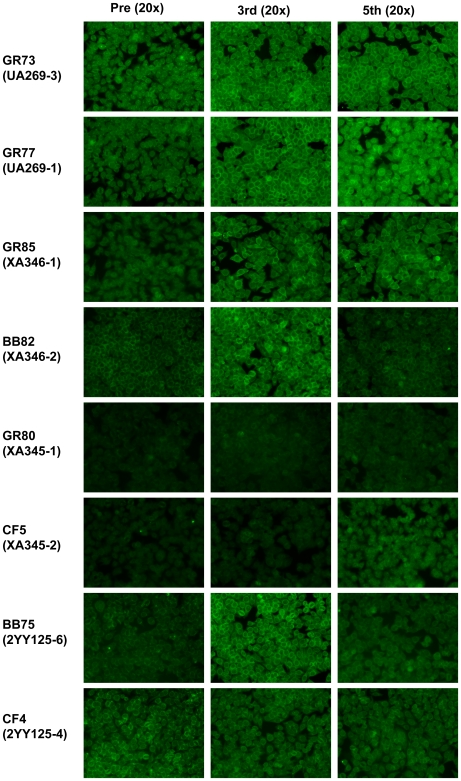
ANA indirect immunofluorescence. Examples of immunofluorescent staining results using pre and post-immune sera from pairs of littermates selected from GR-peptide, BB control and adjuvant control (CF) animals.

Figure 2 shows that in contrast to anti-dsDNA responses, most pre-immune sera assayed by ELISA already had background anti-nuclear antibodies (ANA), anti-Sm and anti-RNP. All post-immunization responses are plotted as ΔOD; the OD at 450 in post-boost sera minus the OD at 450 in pre-immune sera (these background values are shown separately in light blue). The same animal (CF5) that developed anti-dsDNA after the 5^th^ boost also developed an elevated positive anti-RNP response at that time. As observed in earlier studies [5], [9], ANA ELISA values increased in most of the peptide immunized (GR) and backbone immunized (BB) control animals. Increases were also observed in four of the seven CF adjuvant control animals. The contribution of adjuvant treatment to autoantibody responses in this model was raised and discussed in our previous paper [9]. Not only do elevated leukocyte levels typical of chronic inflammatory responses occur due to the immunization protocol using CFA and IFA (Table S1), but in some of these selectively bred rabbits, whether exposed to peptide GR, BB or CF alone, also produce ANA measured by both ELISA and indirect immunofluorescence on HEp-2 cells (Figure 3 and Table 3).

Figure 3 shows examples of test results for ANA detected by indirect immunofluorescence on HEp-2 cells, in pre-immune and post immune sera from litter-mate pairs representing four GR- two BB- and two CF-immunized animals. Fluorescent binding patterns were compared with pictures in [21] and with reference pictures provided with the Antibodies, Inc. kit. Patterns shown in Figure 3 include homogeneous and peripheral nuclear, speckled, cytoplasmic, and nucleolar staining. We previously noted that ANA indirect immunofluorescent staining patterns using sera from BB immunized animals generally differed from those of GR immunized animals, but in those studies the contribution of adjuvant alone was not evaluated [5], [9]. A retrospective study of 690 sera from 58 SLE patients and1187 sera from other patient controls, reported that sera of SLE patients with high levels of anti-dsDNA did not necessarily exhibit homogeneous staining but were also found associated with speckled or nucleolar patterns [22]. They reported that patterns of ANA staining with sera of SLE patients were typical for a particular patient [22]. We therefore chose examples of results of ANA indirect immunofluorescence tests of pre-immune and post immune sera from pairs of littermates (Figure 3). Littermates UA269-3 and -1 were both GR immunized (GR73 and GR77). The peripheral nuclear staining patterns of both appeared similar at 3^rd^ boost and GR73 remained similar at 5^th^ boost. However staining intensity with 5^th^ boost serum of GR77 increased and the pattern appeared speckled. GR85 and BB82 (XA346-1 and 2) exhibited distinct staining patterns with GR85 showing some cytoplasmic and peripheral nuclear staining not seen in its BB-immunized BB82; littermates GR80 and CF5 (XA345-1 and -2) both exhibited only minimal ANA staining although CF5 that produced some anti-dsDNA after the fifth boost also developed increased ANA fluorescence with some nucleolar staining. Finally, background pre-immune ANA peripheral nuclear staining with sera of littermates BB75 and CF4 (2YA125-6 and -4) remained the same although it became slightly more intense in BB75 after the 3^rd^ boost. As with human SLE patients, we found IFA patterns reflecting responses to one or more autoantigens in different rabbits.

### Analyses of BAFF, BR3 and TACI by flow cytometry

**Figure 4:**
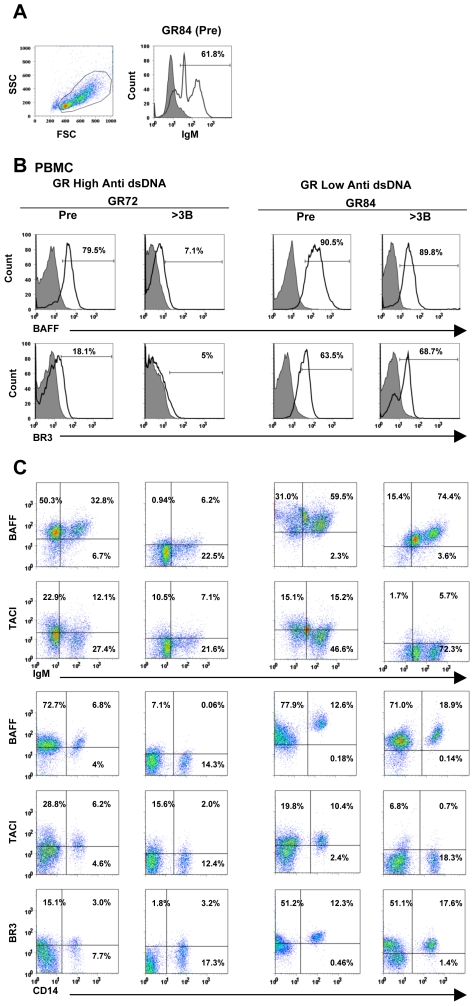
Typical profiles from flow cytometry measurements. A. PBMCs were gated based on forward and side scatter (FSCxSSC) profiles (left panel). IgM^+^ cells in peripheral blood can be divided into IgM^high^ and IgM^low^ cell populations (right panel). B. Representative plots from pre-immune (Pre) and post 3^rd^ boost (>3B) analyses of GR anti-dsDNA high responder (GR72) and low responder (GR84) rabbits. The histograms show the staining of total PBMC for BAFF and BR3 and the control biotin-labeled normal goat IgG for BAFF normal goat IgG for BR3 (shaded). C. Representative dot plots from GR anti-dsDNA high responder (GR72) and low responder (GR84). The plots showed the staining of BAFF and TACI on the surface of peripheral blood IgM^+^ cells and of BAFF, TACI and BR3 on CD14^+^ cells.

The set of data collected is tabulated as supplemental online information (Table S2). Examples of flow cytometry (FACS) profiles are shown in Figure 4. In the table, “total cells” refers to all live cells in the lymphocyte monocyte gate (Figure 4A left panel). Cells were gated on side scatter×forward scatter (SSC×FSC) profiles to include both small and large lymphocytes as well as monocytes but to exclude red blood cells and granulocytes. We analyzed CD14^+^ cells because in our previous report on BAFF and BR3 in normal rabbits [19], we showed that in PBMC, it was the CD14^+^ cells that expressed high levels of BAFF mRNA. In the example shown in Figure 4A, right panel, IgM staining of the gated cells from pre-immune PBMC of rabbit GR84 shows that two positive cell populations were observed (IgM^high^ and IgM^low^). In Table S2, percentages and median fluorescent intensities (MFI) shown are of IgM^+^ (IgM^high^ and IgM^low^) cells because it is not always possible to clearly delineate the two populations. Percentages of cells positive by FACS and their median fluorescence intensities shown permit comparisons of staining results on pre-immune samples (Pre) with results on samples after 3rd and 5th boosts. Colors in Table S2 highlight values that increased (orange), or decreased (blue), compared to pre-immune values. In Figure 4B, the histograms show the staining of total PBMC from pre-immune and post 3^rd^ boost of GR-immunized anti-dsDNA high responder (GR72) and low responder (GR84) for BAFF or BR3 and the control antibody. In both rabbits percentages of BAFF and BR3 positive cells decreased or remained essentially unchanged (Table S2). Figure 4C (upper two rows) shows representative results of double staining for BAFF or TACI on the surface of IgM^+^ cells from PBMC and for BAFF, TACI or BR3 on CD14^+^ cells from the same high (GR72) and low (GR84) responders (lower three rows). Percentages of cells in individual quadrants are indicated. The percentages of total BR3^+^ PBMC and BR3^+^ CD14^+^ cells in PBMC detected by FACS increased in two of four high anti-dsDNA responder animals (GR77 and GR80). Median fluorescence intensities (MFI) of staining, although very low, also increased on cells of the same two animals (Table S2).

**Figure 5:**
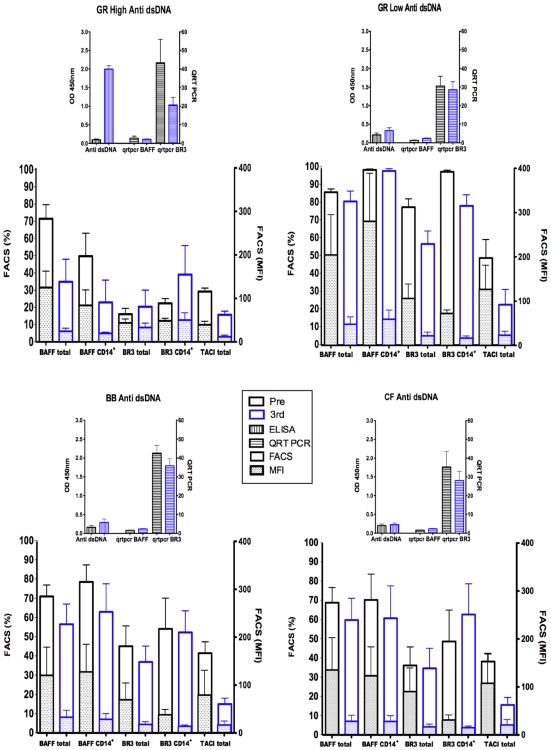
Summary of BAFF and BR3 mRNA expression by Q-PCR, BAFF, BR3, and TACI expression by FACS. Plotted are means and SEM of results comparing high and low anti-dsDNA responders of GR- immunized with BB- and CF-immunized rabbits (anti-dsDNA OD at 450 nm as determined by ELISA). This overview summarizes results of measurements of pre-immune (Pre) and post boost 3 (3^rd^) BAFF and BR3 mRNA in total PBMC by Q-PCR [in Table 3, the unit number showing relative mRNA levels in each sample was determined as a value of mRNA normalized against peptidylprolyl isomerase A (PPIA)]. The figure also shows means and SEM of results of FACS analyses of percentages of cells and median fluorescence intensities (MFI) of BAFF, TACI and BR3 (Table S2 and Figure 4) on total PBMC, or CD14^+^ cell populations from PBMC (the bars are not stacked). In the GR high anti-dsDNA responders, three way ANOVA with repeated measures indicated significant decreases in percentages and MFI of BAFF^+^ cells among total PBMC (p 0.015 and 0.0002 respectively) as well as MFI on CD14^+^ cells (p 0.012). Decreased percentage of BAFF^+^ CD14^+^ cells was near significance (p 0.06). Decreased percentages of TACI^+^ cells among PBMC were significant or near significant in GR low, BB and CF groups (p 0.03, 0.013, 0.09 respectively); decreased TACI MFI were also near significant or significant (0.06, 0.07 and 0.03 respectively).

In order to provide an overview of FACS analyses of BAFF, TACI and BR3 on total PBMC, or CD14^+^ cell populations from PBMC (Table S2 and Figure 4) and results of measurements of BAFF and BR3 mRNA in total PBMC by Q-PCR (Table 3), Figure 5 shows results pre-immunization and after the third boost grouped according to high and low anti-dsDNA responses of GR-immunized compared with BB- and CF- immunized animals. Percentages of BAFF positive cells and MFI generally remained unchanged or decreased in all the immunized groups but a decrease was most consistent in the high anti-dsDNA responder animals. Another consistent change observed by FACS staining was in TACI. Percentages and MFI of TACI staining of total PBMC decreased in most of the immunized rabbits in all groups.

### Q-PCR analyses of BAFF, BR3 and B2M expression

BR3 mRNA levels decreased after the third boost in six of eight GR-immunized rabbits (Table 3 and Figure 5). BR3 mRNA levels in BB and CF immunized also decreased slightly in most animals. The levels of BAFF mRNA in PBMC pre-immunization and in cells collected after the 3rd boost generally remained low and unchanged (Table 3 and Figure 5). Two rabbits (GR84 and 85) that exhibited low anti-dsDNA responses had increased BAFF mRNA after the 3rd boost. In each group, increased levels of mRNA encoding B2M were found in half of the immunized rabbits after the third or fifth boost (Table 3).

Figure 5 provides an overview that summarizes results of measurements of pre-immune (Pre) and post boost 3 (3^rd^) BAFF and BR3 mRNA in total PBMC by Q-PCR (Table 3), FACS analyses of percentages of cells and median fluorescence intensities (MFI) of BAFF, TACI and BR3 on total PBMC, or CD14^+^ cell populations from PBMC (Table S2 and Figure 4). In the GR high anti-dsDNA responders, three way ANOVA with repeated measures indicated significant decreases in percentages and MFI of BAFF^+^ cells among total PBMC (p 0.015 and 0.0002 respectively) as well as MFI on CD14^+^ cells (p 0.012). Decreased percentage of BAFF^+^ CD14^+^ cells was near significance (p 0.06). Decreased percentages of TACI^+^ cells among PBMC were significant or near significant in GR low, BB and CF groups (p 0.03, 0.013, 0.09 respectively); decreased TACI MFI were also near significant or significant (p 0.06, 0.07 and 0.03 respectively).

### Summary of autoantibody responses and pedigree

**Figure 6:**
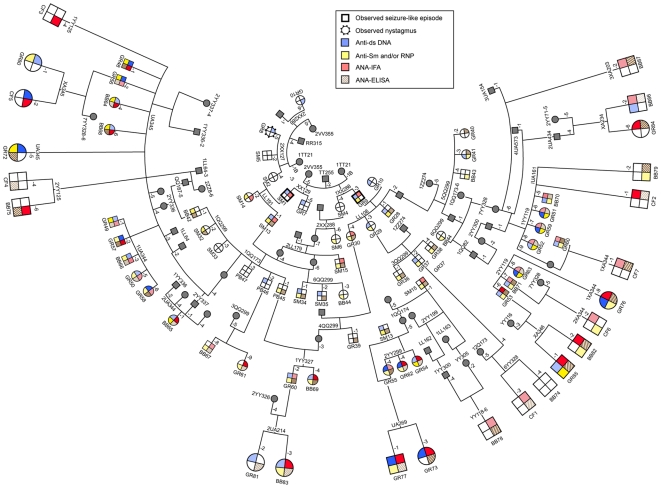
Pedigree and summary of autoantibody responses. An overview of key ancestors, autoantibody responses, and the relationship of males (large squares) and females (large circles) in the current (group 6) to rabbits in 5 earlier groups is shown. Colors in the four quadrants indicate postimmunization elevations of levels of anti-dsDNA (blue, upper left), anti-Sm and/or anti-RNP (yellow, lower left), ANA by IFA (red upper right) and ANA by ELISA (brown diagonal lines, lower right). For the previous group 5 [9] and present group 6 animals, darker colors indicate high autoantibody responses (Tables 3).

A pedigree showing summaries of the responses of selected animals from five previous immunization groups 1–5 was published [9]. Figure 6 shows the present group 6 added on to ancestors reported previously [5], [9] and summarizes their autoantibody responses. Responders such as GR72, 73, 76,77 and 85 can trace relationships in their pedigree back to key founders including rabbits that exhibited seizures (SM1, GR9), and high responder males including SM13 and SM15 [5]. The trend toward more consistent autoantibody production observed in group 5 [9], continues here. Immunization of relatives of GR peptide immunized animals with Freund's complete followed by incomplete adjuvant (CF group) or with backbone alone (BB group) did elicit some production of autoantibodies. This suggests that in this line of rabbits, some have developed susceptibility to development of autoantibodies regardless of the immunogen. However, inclusion of the eight copies of GR peptide on the backbone of branched polylysine (GR-MAP-8) more consistently elicited autoantibody production.

## Discussion

The rabbit has long been a model for human autoimmune and infectious diseases. It is now increasing in importance because the genetics of the immune system is well defined, the entire rabbit genome has been sequenced and a high quality draft assembly (7× coverage) was completed in April 2009 (GenBank Accession Number AAGW02000000). The assembly of the 2× coverage completed in 2005 plus the 7× trace archive already provided useful information that has contributed to the present report and the previous one that described expression and localization of rabbit BAFF and its specific receptor BR3 in cells and tissues of normal rabbits [19]. Human disease models developed in non-inbred but genetically defined animals are better able to reflect the complexities of those human diseases with familial patterns of inheritance due to variants of genes at numerous loci with potential to contribute to the disease phenotype. Systemic Lupus Erythematosus and other autoimmune diseases are in this category. Our model of SLE that elicits autoantibodies after immunization with a peptide from the NMDA glutamate receptor, can provide a means for development of further insights into neuropsychiatric lupus, and pursuit of therapeutic targets.

Limitations were imposed upon the work presented here because there are currently no rabbit-specific reagents for BAFF and its receptors available. We therefore had to discover and utilize cross-reactive antibodies. This decreased sensitivity of analyses and quantitation by FACS. Fortunately, sequences of rabbit BAFF, BR3 and B2M allowed us to conduct successful quantitative PCR measurements of mRNA levels in PBMC.

Just as there are known differences between mice and humans in details of the effects of BAFF and its receptors on B-cell subsets, and their regulated development, there are likely to be additional species-specific aspects of the complex regulation of rabbit B- cell development including involvement of BAFF, BR3, TACI, APRIL and BCMA. Although BAFF is essential for development and maintenance of most B-cells, CD5-positive B-1 cells develop in mice lacking BAFF or BR3 [23], [24]. However, it has more recently been observed that BAFF does stimulate mouse B1 B cells (25). Although only a small proportion of mouse and human B cells are CD5^+^, in rabbits the majority of B cells as well as T cells are CD5^+^ [26], [27]. However, rabbit CD5^+^ B cells are not functional equivalents of murine B-1 B-cells and some features of rabbit CD5^+^ B cells differ from human and mouse [28]. It also appears that the CD5 associated with rabbit B- and T-lymphocytes differs [29]. Immunohistochemical studies of spleens from normal [19] and immunized rabbits (data not shown) detected BAFF staining on large CD4+ T cells IgM^+^ B cells, but we did not detect differences in CD5 staining in immunized compared to normal rabbits.

Another major limitation has been our inability to detect serum BAFF with currently available cross-reacting reagents. We previously demonstrated that BAFF mRNA was not detectable in IgM^+^ B cells from normal rabbit PBL suggesting that BAFF detected by flow cytometry on the B cells was probably soluble BAFF bound to BAFF receptors [19]. Normal human peripheral blood B cells and tonsillar naive and memory B cells were found to have pre-bound BAFF although tonsillar activated B cells did not [30]. Higher occupancy of BR3 by BAFF was reported to correlate with disease activity in man [31]. The BAFF staining on B cells in peripheral blood and spleens from rabbits producing autoantibodies may either reflect BAFF produced by the B cells of rabbits with lupus-like autoantibodies or again be bound through BAFF receptors on B cells. In those GR high anti-dsDNA rabbits in which BAFF detected on PBMC decreased significantly, perhaps less pre-bound BAFF was present on BR3 or other receptors. Although surface expression of BAFF, BR3 and TACI detected by flow cytometry decreased on PBMC after immunization and boosting in most animals, two GR high anti-dsDNA rabbits (GR76 and GR77) developed higher percentages of BAFF^+^ CD14^+^, BR3^+^ CD14^+^, as wells as BR3^+^ cells among total PBMC. Conceivably occupancy of BR3 by soluble BAFF may block access to BR3 by anti-BR3 antibody on large activated PBMC. CD14 is found on the surface of peripheral blood monocytes and macrophages, acts as a co-receptor for detection of bacterial lipopolysaccharide (LPS), and mediates innate immune responses to LPS leading to cytokine secretion and inflammatory responses [32]. Our FACS data also showed that CD14 is also found on large IgM^low^ rabbit B cells (data not shown).

We observed consistently lower TACI percentages and MFI on total PBMC by FACS in most injected animal after their third boosts. It could be that in the rabbit, TACI also plays a negative regulatory role as observed in mice [33]. Other functions of TACI in mice include regulation of the T-independent humoral responses [34]. More complex roles for TACI in human peripheral B-cell development, class switch recombination and terminal differentiation were suggested by discoveries of TACI deficient patients [35], [and reviewed in 36]. Since TACI appears to function like a negative regulator of B cells in mice and humans, in this rabbit model of SLE, perhaps the decrease in TACI allows the autoreactive B cells to escape selection/regulation and thereby, produce auto-antibodies.

We were unable to address roles for potential rabbit homologs of APRIL and BCMA. APRIL is the TNF family member most closely related to BAFF. APRIL also binds TACI and BCMA and is expressed by lymphoid cells and some tumor cells. Recently Ingold et al [37] proposed that proteoglycans such as syndecan are APRIL-specific receptors that bind APRIL, induce oligomerization prior to the interaction of APRIL with TACI or BCMA to initiate survival signals. Additional complexities are likely due to different forms (alternatively spliced, trimer, 60-mer of BAFF plus heteromeric forms of BAFF with APRIL) interacting with the different receptors for BAFF. Bossen et al [38] reported that although BAFF-R and TACI can provide B cells with similar signals, only BAFF-R, but not TACI, can respond to soluble BAFF trimer. They found that BAFF 60-mer was more than 100-fold more active than BAFF 3-mer for the activation of multimerization-dependent signals.

It is hoped that the limitations we encountered in working with few available reagents to detect cells and proteins of the rabbit immune system will diminish and companies will produce more rabbit-specific reagents because there is an increasing community of researchers who use rabbits for research on autoimmune, infectious, neurological, cardiovascular, ocular and other diseases. Using the new NIH research portfolio online reporting tool (RePORT) http://projectreporter.nih.gov, 424 current projects match the search criteria: “Rabbit, Fiscal Year: Current Projects”. See also, “The Rabbit in Immunology & Infectious Disease Research” at: http://www3.niaid.nih.gov/LabsAndResources/resources/ri/.

The differences we have observed between individual rabbits mirror similar heterogeneity of populations of lupus patients. Large patient populations were required in order to detect some correlations between BAFF expression, autoantibody production, and disease status [14]–[16].

This study confirms and extends evidence for a genetic contribution to autoantibody responses in the rabbit SLE model we developed. In spite of the complexities of interactions of multiple receptors and ligands in the BAFF system discussed above, future gene expression analyses of cells and tissues from rabbits with induced autoantibody production may reveal dysregulated pathways that permit autoantibody-producing B cells to survive.

## Supporting Information

Table S1 (0.12 MB DOC)

Table S2 Summary of FACS data
(0.11 MB PDF)
