# Supplementary material for: Investigations of a Rabbit (Oryctolagus cuniculus) Model of Systemic Lupus Erythematosus (SLE), BAFF and Its Receptors
Source: PLoS One. 2009 Dec 30;4(12):e8494. doi: 10.1371/journal.pone.0008494 (PMC2794537; doi:10.1371/journal.pone.0008494)
Supplement: Table S1 — (0.12 MB DOC) [file pone.0008494.s001.doc]

**Table S1. Total White Blood Cell (WBC), Monocyte, Neutrophil, Eosinophil and Basophil Counts**

WBC Neutrophils Lymphocytes Monocytes Eosinophils Basophils

| Rabbit ID | | Pre | | 3rd | 5th | | | | Pre | | | 3rd | | 5th | | | Pre | | | | 3rd | | | 5th | | | Pre | | 3rd | | | | 5th | | Pre | | | 3rd | | | 5th | | Pre | | | 3rd | 5th | |  | |
| --- | --- | --- | --- | --- | --- | --- | --- | --- | --- | --- | --- | --- | --- | --- | --- | --- | --- | --- | --- | --- | --- | --- | --- | --- | --- | --- | --- | --- | --- | --- | --- | --- | --- | --- | --- | --- | --- | --- | --- | --- | --- | --- | --- | --- | --- | --- | --- | --- | --- | --- |
| UA345-5 (GR72) | | 8.1 | | **11.8** | | -- | | 3.24 | | | 5.66 | | | | -- | | | 4.05 | | 4.96 | | | -- | | | 0.16 | | | | 0.35 | | -- | | | | **0.24** | | **0.24** | | -- | | | 0.41 | | **0.59** | | -- | | |  |
| UA269-3 (GR73) | | 5.9 | | 9.3 | | 9.1 | | 1.30 | | | 3.63 | | | | 3.19 | | | 4.13 | | 4.28 | | | 4.90 | | | 0.06 | | | | **0.65** | | 0.18 | | | | **0.12** | | **0.28** | | 0.18 | | | 0.30 | | 0.47 | | **0.64** | | |  |
| 2YY119-9 (GR76) | | 6.8 | | **14.8** | | -- | | 1.77 | | | **11.25** | | | | -- | | | 4.56 | | 3.11 | | | -- | | | 0.20 | | | | **0.44** | | -- | | | | **0.14** | | 0 | |  | | | 0.14 | | 0 | | -- | | |  |
| UA269-1 (GR77) | | 7.3 | | 6.9 | | **11.8** | | 1.61 | | | 3.93 | | | | 4.01 | | | 5.11 | | 2.76 | | | 6.84 | | | 0.15 | | | | 0.21 | | **0.35** | | | | 0.07 | | 0 | | **0.12** | | | 0.37 | | 0 | | 0.47 | | |  |
| XA345-1 (GR80) | | 6.5 | | 9.9 | | 8.5 | | 1.82 | | | 4.36 | | | | 3.23 | | | 3.84 | | 4.65 | | | 4.08 | | | **0.39** | | | | 0.20 | | **0.60** | | | | **0.13** | | **0.20** | | **0.17** | | | 0.33 | | 0.50 | | 0.43 | | |  |
| 2UA14-2 (GR81) | | 4.1 | | **10.1** | | **11.7** | | 1.52 | | | 4.24 | | | | 5.03 | | | 2.26 | | 4.55 | | | 5.50 | | | 0.29 | | | | **0.71** | | **0.59** | | | | 0 | | 0.10 | | **0.12** | | | 0.04 | | **0.51** | | 0.47 | | |  |
| XA234-6 (GR84) | | 5.9 | | **11.1** | | 8.7 | | 1.18 | | | 4.44 | | | | 3.65 | | | 4.07 | | 5.22 | | | 3.83 | | | 0.30 | | | | **0.44** | | **0.52** | | | | **0.12** | | **0.33** | | **0.26** | | | 0.24 | | **0.67** | | 0.44 | | |  |
| XA346-1 (GR85) | | 8.4 | | **15.3** | | **18.7** | | 2.69 | | | **7.65** | | | | **8.79** | | | 5.29 | | 6.12 | | | 8.04 | | | 0.25 | | | | **1.07** | | **0.94** | | | | 0.08 | | **0.31** | | **0.37** | | | 0.08 | | 0.15 | | **0.56** | | |  |
| 6YY328-4 (BB74) | | **11.3** | | **13.9** | | -- | | 4.63 | | | **7.92** | | | | -- | | | 5.20 | | 4.17 | | | -- | | | **0.57** | | | | **0.83** | | -- | | | | **0.23** | | **0.28** | | -- | | | **0.68** | | **0.70** | | -- | | |  |
| 2YY125-6 (BB75) | | 8.7 | | **11.8** | | **11.2** | | 2.00 | | | 4.96 | | | | 5.38 | | | 6.09 | | 5.31 | | | 4.37 | | | 0.17 | | | | **0.71** | | **0.67** | | | | **0.17** | | **0.24** | | **0.22** | | | 0.26 | | 0.59 | | 0.56 | | |  |
| YY118-6 (BB78) | | 6.4 | | **11.1** | | -- | | 1.86 | | | 6.22 | | | | -- | | | 3.71 | | 4.00 | | | -- | | | **0.38** | | | | **0.89** | | -- | | | | **0.13** | | 0 | | -- | | | 0.32 | | 0 | | -- | | |  |
| 1UA161-2 (BB79) | | 8.1 | | **11.4** | | **14.7** | | 1.70 | | | **8.78** | | | | **7.06** | | | 5.75 | | 1.25 | | | 6.03 | | | 0.16 | | | | **1.14** | | **0.74** | | | | **0.16** | | 0 | | **0.29** | | | 0.32 | | 0.23 | | 0.59 | | |  |
| XA346-2 (BB82) | | 6.6 | | **13.9** | | **14.5** | | 1.58 | | | **7.23** | | | | **7.25** | | | 4.62 | | 5.14 | | | 6.09 | | | 0.20 | | | | **0.97** | | **0.58** | | | | 0.07 | | **0.14** | | **0.29** | | | 0.13 | | 0.42 | | 0.29 | | |  |
| 2UA14-3 (BB83) | | 6.9 | | **11.7** | | 9.8 | | 1.52 | | | 6.20 | | | | 4.02 | | | 4.83 | | 3.98 | | | 4.61 | | | 0.14 | | | | **0.82** | | **0.49** | | | | 0.07 | | **0.12** | | **0.20** | | | 0.35 | | **0.59** | | 0.49 | | |  |
| XA234-2 (BB86) | | 6.3 | | 9.9 | | **10.3** | | 1.58 | | | 3.37 | | | | 3.14 | | | 4.41 | | 5.84 | | | 6.39 | | | 0.13 | | | | 0.30 | | 0.21 | | | | 0.06 | | 0.10 | | 0.10 | | | 0.13 | | 0.30 | | 0.41 | | |  |
| 3XA203-2 (BB87) | | 8.0 | | 9.8 | | **10.8** | | 1.60 | | | 4.31 | | | | 4.32 | | | 5.76 | | 4.41 | | | 5.08 | | | 0.16 | | | | **0.59** | | **0.65** | | | | 0.08 | | 0.10 | | **0.54** | | | 0.40 | | 0.39 | | 0.22 | | |  |
| 6YY328-3 (CF1) | | **11.0** | | **15.0** | | -- | | 2.75 | | | **8.10** | | | | -- | | | **7.15** | | 5.10 | | | -- | | | 0.22 | | | | **0.75** | | -- | | | | **0.22** | | **0.30** | | -- | | | **0.66** | | **0.75** | | -- | | |  |
| 1UA161-1 (CF2) | | 7.4 | | **16.2** | | **14.6** | | 2.20 | | | **9.07** | | | | **8.61** | | | 4.29 | | 5.18 | | | 4.53 | | | **0.37** | | | | **1.13** | | **0.73** | | | | **0.15** | | **0.16** | | **0.15** | | | 0.37 | | **0.65** | | **0.58** | | |  |
| 1YY125-4 (CF3) | | 9.1 | | 6.3 | | -- | | 2.28 | | | 3.47 | | | | -- | | | 6.01 | | 2.27 | | | -- | | | **0.46** | | | | **0.57** | | -- | | | | 0.09 | | 0 | | -- | | | 0.27 | | 0 | | -- | | |  |
| 2YY125-4 (CF4) | | 5.5 | | 4.7 | | 6.6 | | 1.32 | | | 2.63 | | | | 2.31 | | | 3.41 | | 1.79 | | | 3.70 | | | **0.33** | | | | 0.28 | | 0.13 | | | | **0.17** | | 0 | | **0.13** | | | 0.28 | | 0 | | 0.33 | | |  |
| XA345-2 (CF5) | | 6.4 | | 9.1 | | 8.3 | | 2.30 | | | 4.91 | | | | 4.15 | | | 3.20 | | 2.82 | | | 2.91 | | | **0.45** | | | | **0.64** | | **0.58** | | | | **0.13** | | **0.27** | | **0.25** | | | 0.32 | | 0.46 | | 0.42 | | |  |
| 2XA344-2 (CF6) | | 7.2 | | **17.0** | | **15.0** | | 2.88 | | | **8.50** | | | | **7.50** | | | 3.60 | | 6.46 | | | 5.25 | | | 0.22 | | | | **0.85** | | **0.90** | | | | **0.14** | | **0.34** | | **0.75** | | | 0.36 | | **0.85** | | **0.60** | | |  |
| 1XA344-1 (CF7) | | 5.3 | | **12.3** | | **12.4** | | 1.48 | | | 5.29 | | | | 5.58 | | | 3.23 | | 5.90 | | | 5.46 | | | **0.32** | | | | **0.49** | | **0.87** | | | | 0.05 | | **0.12** | | **0.12** | | | 0.21 | | 0.49 | | 0.37 | | |  |
|  | Mean  SD | | 7.30  1.71 | 11.40  3.17 | | | 11.60  3.12 | | | 2.00  0.79 | | | 5.90  2.23 | | | 5.10  2.02 | | | 4.50  1.13 | | | 4.30  1.41 | | | 5.00  1.28 | | | 0.26  0.13 | | | 0.65  0.29 | | | 0.57  0.25 | | | 0.12  0.06 | | 0.16  0.12 | | | 0.25  0.17 | | 0.30  0.15 | | 0.40  0.26 | | 0.46  0.12 | | |

***Values in bold face are above the normal reference ranges (x103/μl) for White Blood Cells 4-10; Neutrophils,1.2-7.0; Lymphocytes,1.2-7.0; Monocytes,0-0.30; Eosinophils,0-0.10; Basophils,0-0.50.**
