# Supplementary material for: Investigations of a Rabbit (Oryctolagus cuniculus) Model of Systemic Lupus Erythematosus (SLE), BAFF and Its Receptors
Source: PLoS One. 2009 Dec 30;4(12):e8494. doi: 10.1371/journal.pone.0008494 (PMC2794537; doi:10.1371/journal.pone.0008494)
Supplement: Table S2 — Summary of FACS data (0.11 MB PDF) [file pone.0008494.s002.pdf]

Table S2 Summary of FACS data*

BAFF

	Total (FACS)			Total (MFI)			IgM High+Low(FACS)			IgM High+Low (MFI)			CD14+ (FACS)			CD14+ (MFI)		
Rabbit	Pre	3rd	5th	Pre	3rd	5th	Pre	3rd	5th	Pre	3rd	5th	Pre	3rd	5th	Pre	3rd	5th
UA345-5 (GR72)	79.46	7.14		41.05	4.10		83.12	24.95		48.26	6.1		62.84	0.45		27.38	3.10	
UA269-3 (GR73)	89.25	24.39	64.62	52.8	5.67	18.11	93.7	32.73	90.64	57.77	7.37	30.78	80.4	0.44	93.85	43.32	4.00	31.34
2YY119-9 (GR76)	51.8	39.47		12.52	3.11		67.73	48.95		18.94	5.5		22.1	44.69		5.83	5.94	
UA269-1 (GR77)	65.42	68.69	51.10	18.60	11.34	4.30	72.65	71.63	70.89	20.54	12.8	6.61	33.74	46.03	61.35	7.37	6.40	4.70
XA345-1 (GR80)	81.73	71.61	69.30	49.60	22.00	73.20	93.69	92.99	85.86	62	32.7	22.4	98.66	97.02	95.07	101.0	26.60	34.10
2UA14-2 (GR81)	85.8	68.89	69.03	44.50	19.20	66.10	94.99	86.53	84.48	51.7	25.2	19.8	98.79	94.01	91.61	96.80	19.60	31.50
XA234-6 (GR84)	90.45	89.81	71.33	381.0	61.40	75.60	96.36	95.34	84.82	182	32.8	22.1	98.59	99.24	91.68	389.0	97.20	53.40
XA346-1 (GR85)	84.12	91.12	71.49	342.0	88.90	64.70	91.04	97.31	88.56	161	46.3	27.1	96.52	99.76	89.7	534.0	93.00	45.40
6YY328-4 (BB74)	58.01	9.98		26.66	3.34		81.01	15.71		40.32	4.96		76.87	0.33		38.89	3.72	
2YY125-6 (BB75)	68.24	16.31	58.07	31.66	4.41	15.26	84.74	28.31	79.58	44.51	6.32	31.91	78.25	0.32	87.95	37.86	3.52	24.8
YY118-6 (BB78)	65.56	58.00		16.70	7.70		75.67	74		19.81	10.75		43.48	61.16		9.60	9.14	
1UA161-2 (BB79)	39.57	56.7	44.83	8.90	7.70	3.31	47.63	77.5	69.77	10.55	13.1	6.21	38.23	64.47	76.88	8.74	9.82	6.67
XA346-2 (BB82)	82.43	64.59	66.99	49.20	21.50	82.20	96.28	92.4	90.18	65.1	33.4	24.2	97.22	83.14	78.50	76.60	15.90	22.10
2UA14-3 (BB83)	89.8	67.09	76.93	48.50	21.70	100.0	95.52	86.76	87.76	53.6	30.5	20.7	98.42	94.35	90.59	83.70	21.00	30.80
XA234-2 (BB86)	87.52	90.31	79.73	383.0	100.0	90.20	93.63	96.77	91.26	147	37.3	28.4	97.64	99.92	91.59	450.0	86.10	51.40
3XA203-2 (BB87)	76.83	89.20	74.61	397.0	96.20	78.50	79.11	97.34	85.76	99.1	37.2	20.1	98.19	99.76	90.12	314.0	78.60	46.70
6YY328-3 (CF1)	54.29	12.68		23.50	3.28		76.68	18.43		35.55	4.37		84.38	0.36		46.98	3.49	
1UA161-1 (CF2)	89.92	23.97	47.85	62.08	2.94	10.94	95.05	51.06	93.15	66.71	10.9	34.29	69.18	0.30	84.08	32.20	2.67	21.87
1YY125-4 (CF3)	36.84	70.28		8.82	10.46		41.05	79.19		9.22	12.19		25.3	53.83		5.42	7.43	
2YY125-4 (CF4)	50.78	73.62	63.29	11.76	10.65	5.57	57.67	76.93	79.08	12.86	11.14	7.23	16.3	72.41	56.79	4.87	11.14	4.70
XA345-2 (CF5)	81.69	62.75	67.01	51.60	15.80	90.30	94.19	87.64	86.76	65.6	25.2	21.7	98.97	98.14	93.58	88.50	24.70	31.80
2XA344-2 (CF6)	82.16	86.19	73.80	413.0	82.40	101.0	88.42	93.54	84.25	109	32.3	24.8	99.3	99.69	81.40	260.0	67.00	37.50
1XA344-1 (CF7)	85.4	88.77	68.60	377.0	72.00	73.80/	91.86	96.94	89.01	143	30.6	22	97.78	99.72	93.12	425.0	79.10	54.50

BR3

	Total (FACS)			Total (MFI)			CD14+ (FACS)			CD14+ (MFI)		
Rabbit	Pre	3rd	5th	Pre	3rd	5th	Pre	3rd	5th	Pre	3rd	5th
UA345-5 (GR72)	18.11	5.00		12.9	4.01		28.17	15.60		15.90	6.43	
UA269-3 (GR73)	24.78	2.39	34.53	16.4	3.96	7.10	21.55	5.35	30.00	12.60	4.46	6.15
2YY119-9 (GR76)	9.96	36.08		7.13	11.90		15.11	61.28		9.12	17.70	
UA269-1 (GR77)	11.34	38.14	50.75	7.26	13.30	4.85	24.82	74.08	69.67	10.80	21.6	5.60
XA345-1 (GR80)	84.33	49.43	59.46	58.2	7.48	115.0	99.01	65.74	91.5	95.30	7.36	53.90
2UA14-2 (GR81)	81.71	39.41	61.53	50.7	6.73	105.0	97.89	69.51	82.18	71.70	8.61	46.30
XA234-6 (GR84)	63.52	68.68	50.81	189	35.1	124.0	96.38	92.53	93.45	61.10	23.60	68.00
XA346-1 (GR85)	79.09	68.43	82.37	127	37.7	108.0	95.01	83.79	92.76	60.70	26.70	64.30
6YY328-4 (BB74)	19.27	1.71		11.90	4.25		1.97	3.38		3.36	5.82	
2YY125-6 (BB75)	29.12	5.60	43.37	17.20	5.04	9.78	25.95	13.41	44.99	15.00	7.09	9.64
YY118-6 (BB78)	13.82	42.38		7.68	14.30		14.49	76.68		8.84	21.60	
1UA161-2 (BB79)	11.27	35.03	61.75	8.14	12.80	5.91	7.16	40.9	75.75	7.26	13.80	6.34
XA346-2 (BB82)	86.11	50.27	60.34	50.30	9.18	97.00	99.01	55.71	53.44	70.30	6.73	30.40
2UA14-3 (BB83)	67.91	42.51	55.48	39.10	8.90	136.0	94.05	57.16	85.67	63.20	6.50	52.90
XA234-2 (BB86)	71.9	44.33	65.33	123.0	43.80	130.0	95.8	74.3	92.55	70.90	23.90	70.70
3XA203-2 (BB87)	61.38	73	69.08	296.0	44.10	182.0	94.44	96.27	91.46	64.40	24.10	69.80
6YY328-3 (CF1)	33.21	1.17		18.90	3.62		7.74	1.65		6.70	2.95	
1UA161-1 (CF2)	26.43	0.70	38.69	15.90	4.37	7.48	11.84	0.54	32.2	10.10	4.41	6.25
1YY125-4 (CF3)	7.32	35.34		6.58	12.80		17.32	78.23		9.33	21.10	
2YY125-4 (CF4)	5.44	33.3	55.94	5.66	12.00	6.44	19.73	91.79	98.68	10.20	27.60	12.10
XA345-2 (CF5)	67.69	35.12	54.62	38.8	5.65	118	98.05	85.26	95.02	67.90	12.00	66.40
2XA344-2 (CF6)	46.66	63.9	57.26	332	37.5	141	91.86	91.17	91.06	56.30	20.80	64.30
1XA344-1 (CF7)	66.12	72.69	72.39	215	40.3	159	93.58	89.53	94.82	56.80	17.10	86.10

Table S2 Summary of FACS data (continued)

TACI

	Total (FACS)			Total (MFI)			IgM High+Low (FACS)			IgM High+Low (MFI)			CD14+ (FACS)			CD14+ (MFI)		
Rabbit	Pre	3rd	5th	Pre	3rd	5th	Pre	3rd	5th	Pre	3rd	5th	Pre	3rd	5th	Pre	3rd	5th
UA345-5 (GR72)	34.94	17.58		14.86	4.22		30.61	24.63		11.97	5.78		57.22	13.69		25.25	4.30	
UA269-3 (GR73)	26.23	20.86	63.52	11.24	4.91	16.55	21.39	25.52	73.09	10	6.21	20.35	61.87	11.82	57.10	30.23	3.46	12.19
2YY119-9 (GR76)	29.43	12.41		7.40	1.45		23.1	15.83		7.43	1.67		11.81	8.60		4.00	1.00	
UA269-1 (GR77)	26.3	12.12	34.20	5.73	1.2	2.44	23.27	11.95	37.35	7.4	1.19	2.55	20.57	7.17	27.19	4.78	1.00	1.65
XA345-1 (GR80)	64.13	39.70	38.17	30.8	8.98	76.7	65.92	39.18	34.57	31.1	8.86	9.74	93.29	78.54	87.97	54.00	12.40	29.60
2UA14-2 (GR81)	68.95	34.48	41.14	34.3	8.67	82.6	70.03	40.58	36.71	33.3	10	9.35	87.84	66.58	66.12	43.20	10.70	19.30
XA234-6 (GR84)	30.24	7.40	7.08	223	46.2	36.7	24.56	7.322	6.655	30	4.44	5.3	81.62	3.44	7.29	43.00	6.57	7.77
XA346-1 (GR85)	31.13	7.77	8.00	219	28.3	39.5	24.65	9.173	9.522	31.5	5.09	5.92	67.82	0.75	2.97	34.20	3.70	5.34
6YY328-4 (BB74)	40.02	11.10		16.55	3.5		32.92	15.7		12.98	4.26		62.75	6.83		28.64	2.94	
2YY125-6 (BB75)	33.94	12.51	42.40	14.2	3.96	10.46	28.75	22.12	38.68	11.65	5.83	6.49	80.60	12.49	60.10	40.68	3.34	12.63
YY118-6 (BB78)	30.3	16.54		6.92	1.64		25.67	18.23		7.77	1.67		14.83	7.86		3.60	1.00	
1UA161-2 (BB79)	37.45	12.70	31.15	6.67	1.31	2.19	29.3	14.7	25.46	9.47	1.23	1.9	26.47	6.29	29.16	5.94	1.00	1.85
XA346-2 (BB82)	64.96	25.42	31.08	35.8	7.26	74.4	59.76	28.69	26.63	32.3	8.34	8.26	83.58	48.18	58.52	45.20	7.70	17.30
2UA14-3 (BB83)	69.47	29.58	40.96	34	7.56	107	70.39	32.19	36.7	31	8.4	11.4	91.08	72.91	81.69	51.40	11.10	25.40
XA234-2 (BB86)	23.43	6.82	6.54	79.1	52.9	90.5	19.89	7.002	7.019	33.4	4.51	5.36	82.86	3.27	6.84	51.70	5.98	6.95
3XA203-2 (BB87)	32.33	5.18	5.57	438	55.1	53.7	29.07	5.823	5.643	35.9	4.56	5.08	71.23	1.69	5.74	36.60	4.66	6.39
6YY328-3 (CF1)	35.63	16.49		13.46	2.86		33.13	32.55		12.75	6.98		89.30	9.27		60.98	2.94	
1UA161-1 (CF2)	45.95	16.50	52.42	19.28	2.79	12.3	35.17	32.96	57.83	14.86	7.4	13.95	67.94	3.42	54.92	33.68	2.31	11.44
1YY125-4 (CF3)	30.6	11.85		4.91	1.60		25.3	12.63		6.15	1		10.24	14.82		3.00	1.24	
2YY125-4 (CF4)	32.44	17.11	35.72	7.17	1.84	2.64	30.56	16.32	37.79	9.14	1.61	2.84	45.06	17.65	58.68	10.00	1.46	4.37
XA345-2 (CF5)	58.87	35.95	46.81	27.2	8.75	106.0	57.51	55.56	40	24.7	11.6	10.3	93.74	85.81	85.95	58.70	16.00	28.70
2XA344-2 (CF6)	31.64	4.44	6.46	419	69.10	57.30	33.26	5.246	7.147	39	3.95	5.74	57.67	1.51	5.62	26.90	5.23	6.41
1XA344-1 (CF7)	31.57	6.44	7.02	262	59.10	52.10	26.97	7.133	7.618	32.5	4.65	6.04	68.57	1.67	3.93	36.20	5.14	6.30

*Percentages of total PBMC, IgM High+Low, or $CD14^+$cells positive by flow cytometry (FACS) and their median fluorescence intensities (MFI) showing comparisons of pre-immune samples (Pre) with samples after 3rd and 5th boosts, Colors highlight values that appear to have significantly increased (orange), or decreased (blue), compared to pre-immune values.
